# Psychometric evaluations of the Theoretical Framework of Acceptability (TFA) among adolescent girls and young women in a low-resource setting

**DOI:** 10.21203/rs.3.rs-9271781/v1

**Published:** 2026-04-23

**Authors:** Laban Muteebwa, Patience A. Muwanguzi, Dan Muramuzi, Ivan Ahimbisibwe, Shivan Nuwasiima, Cathbert Tumusiime, Edson Atwine, Fred C. Semitala, Joanita Nangendo

**Affiliations:** Makerere University; Makerere University; Makerere University; Makerere University; Makerere University; Baylor College of Medicine Children’s Foundation-Uganda; Makerere University Business School, Makerere University; Makerere University; Makerere University

**Keywords:** Acceptability, Theoretical Framework of Acceptability, construct validity, Reliability, adolescent girls and young women, low-resource setting, HIV self-testing

## Abstract

**Background:**

The Theoretical Framework of Acceptability (TFA) provides a comprehensive lens for assessing acceptability of healthcare interventions. While the TFA has seven constructs, limited literature exists on its psychometric properties in low-resource settings and in the context of HIV prevention. This study assessed the validity and reliability of HIV self-testing (HIVST) acceptability scores measured using the TFA among adolescent girls and young women (AGYW) in Uganda.

**Methods:**

We enrolled 377 AGYW aged 15–24 years in a cross sectional study and a structured questionnaire was used to collect data. The primary outcome was acceptability of HIVST defined as willingness to accept HIVST services if offered – measured using the seven constructs of the TFA where each was assessed with one 5-level Likert item question. Construct validation of TFA was examined using the exploratory factor analysis (EFA), confirmatory factor analysis (CFA) – through structural equation modelling (SEM), and convergent and divergent validity, while reliability was assessed with the Cronbach’s alpha. Factor extraction was guided by the scree plot, and factor rotation was performed using Oblimin method. Convergent and divergent validity were assessed by correlating the TFA acceptability scores with willingness to use HIVST and perceived HIV risk respectively.

**Results:**

The median age of participants was 20 years (IQR: 18, 22). Although the scree plot suggested that three factors should be extracted, the rotated solution of the EFA yielded a single dominant factor, with all seven items loading strongly onto one latent factor. In the CFA, both SEM and generalized SEM showed consistent direction and strength of association between the TFA domains and the underlying acceptability construct despite the poor SEM global fit indices (RMSEA = 1.224, CFI = 0.173, TLI= −0.103). The correlation between acceptability scores and willingness to use HIVST, and perceived HIV risk was 0.7 and 0.1 respectively. The overall Cronbach’s alpha for the questionnaire items was 0.889.

**Conclusions:**

This study provides empirical evidence supporting the construct validity and reliability of the TFA-measured acceptability scores among AGYW. All seven constructs demonstrated strong loadings on a single latent factor, indicating that the TFA operates as a coherent and unidimensional tool for measuring overall acceptability.

## Background

Acceptability is increasingly recognized and considered during the design, evaluation and implementation phases of public health interventions and strategies. Acceptability refers to the extent to which people delivering or receiving a healthcare intervention perceive it as appropriate, based on their cognitive and emotional responses [1]. Acceptability of an intervention is a reflective outcome, which presents challenges with its measurement. Sekhon and colleagues, (2017) developed a robust Theoretical Framework of Acceptability (TFA) grounded in literature and following acceptable principles of framework development [1]. The TFA has gained broad acceptance in Implementation Science because it provides a coherent structure for prospective, concurrent and retrospective assessment of user experience of interventions [1]. It has been applied in diverse settings, including tuberculosis care [2], maternal health [3-5], and digital health interventions [6, 7]. The TFA has also been used in HIV-related research in Uganda [6, 8]. Owing to the inconsistent use of TFA measurement, Sekhon and colleagues (2022) developed a generic (adaptable) TFA-informed questionnaire to guide the use of the framework [9]. Although it provides a well-articulated conceptual structure of assessing intervention acceptability, empirical evidence regarding the validity and reliability of TFA-based measurement tools remain limited particularly in low and middle income countries (LMICs) and among adolescent populations. The TFA conceptualizes acceptability as a multifaceted construct comprising of seven domains; affective attitude, burden, perceived effectiveness, ethicality, intervention coherence, opportunity costs and self-efficacy [1]. While these domains provide a theoretically grounded structure for understanding how individuals evaluate healthcare interventions, it is necessary to examine whether measurement items derived from the framework accurately capture the underlying construct when applied to different contexts and populations.

In the context of HIV prevention, assessing acceptability is particularly important because the success of new interventions such as HIV self-testing (HIVST) depends not only on their clinical effectiveness but also on whether potential users perceive them as appropriate, feasible and worthwhile to adopt [1, 10]. Adolescent girls and young women (AGYW) remain disproportionately affected by HIV in the Sub-Saharan African region (SSA), and the interventions targeting this sub-population must be both accessible and acceptable to ensure optimal uptake and sustained use. Although the TFA has increasingly been used to explore acceptability of health interventions in various settings, most studies have applied the framework qualitatively or descriptively, with limited attention to its psychometric performance as a quantitative measurement tool [9]. Establishing the measurement properties of TFA-based instruments is therefore essential for ensuring that acceptability scores derived from such tools accurately represent the theoretical construct and can be reliably used to guide implementation research and program design.

Construct validation is a key step in psychometric evaluation, and refers to the extent to which a measurement tool accurately captures the theoretical concept it is intended to measure [11, 12]. For multi-dimensional constructs such as acceptability, exploratory factor analysis (EFA) and the confirmatory factor analysis (CFA) are commonly used to examine whether the observed questionnaire items reflect the underlying latent structure hypothesized by the theoretical framework [13-15]. In this study, EFA was used to assess whether the items derived from the TFA domains collectively represents the latent concept of acceptability of HIVST among AGYW in Uganda. Then the CFA was used to test whether the pre-defined structure fit the observed data. In addition, convergent and divergent validity provide complementary evidence of construct validity. Convergent validity examines whether measures that are theoretically expected to be related are empirically correlated, whereas divergent (discriminant) validity assesses whether measures representing conceptually distinct constructs show weak or negligible correlations [16].

Reliability is another fundamental property of measurement instruments and refers to the degree to which a tool consistently measures an underlying construct across its component items [12]. In psychometric research, internal consistency reliability is commonly assessed using the Cronbach’s alpha which evaluates the extent to which the items in the scale measure the same latent concept [17, 18]. Establishing reliability is particularly important when applying theoretically derived measurement tools in new populations or settings because variations in context, culture or interpretation of questionnaire items may influence the consistency of the responses. Demonstrating adequate reliability therefore strengthens confidence that an instrument provides coherent measurement of acceptability. This study aimed to assess the construct validity and internal consistent reliability of acceptability scores derived from the TFA when applied to measuring acceptability of HIVST among AGYW in Uganda.

## Methods

### Study design and setting

This cross sectional study was conducted among AGYW living in the Kampala Metropolitan Area in Uganda. Kampala Metropolitan Area includes the four most populous districts in Central Uganda including Kampala, Wakiso, Mukono and Mpigi according to the 2024 National census [19]. This is the most urbanized and commercial area in Uganda and includes its Capital City, Kampala, hosting an estimated daytime population of about five million people [20]. This region has one of the highest burden of HIV and consistently reports high numbers of new HIV infections [21]. The HIV prevalence among adults aged 15–49 years (2023) in Kampala, Wakiso, Mpigi and Mukono is 7.4%, 7.2%, 8.2% and 5.3% respectively, all above the national average (2023) of 5.1% [21]. Additionally, Kampala Metropolitan Area also accounted for the largest number of new HIV cases in 2023 (8,805) which threatens the gains on HIV epidemic control [21].

### Study population and sampling procedure

We enrolled 377 AGYW aged 15 to 24 years who had lived in the study area for at least six months, being sexually active (at least one sexual intercourse in the past six months) with an HIV risk score of ≥ 2 (high-risk), using and HIV risk assessment tool used by Ministry of Health and previously used among AGYW in Kampala, Uganda [22, 23], and gave written informed consent (for adults and emancipated minors) or assent for minors in addition to their guardian’s consent. A multistage sampling design was used to select participants, where in the first stage two out of four districts (Kampala and Wakiso) were selected purposively because they had the highest HIV incident cases [21]. At the second stage, two divisions in Kampala city (Makindye and Rubaga divisions) and two municipalities in Wakiso (Entebbe and Nansana) were selected by simple random sampling. At the third stage, the study participants were selected from communities by consecutive sampling technique, but the number selected from each of the study divisions/municipalities were proportionately allocated based on 2024 population projections of AGYW [24].

### Theoretical Framework of Acceptability (TFA)

The TFA was developed by Skehon and colleagues (2017) after a comprehensive literature review and then applied principles of inductive and deductive reasoning to theorize the concept of acceptability [1]. The authors contend that acceptability of an intervention can be assessed prospectively (pre-intervention phase), concurrently (during implementation phase) or retrospectively (after the implementation phase). Particularly for the prospective acceptability, the authors contend that prior to experiencing an intervention, both recipients and providers can form judgements about whether they expect the intervention to be acceptable or unacceptable. Such judgements may be based on the information provided about the intervention. The framework specifies seven distinct constructs of acceptability: affective attitude, burden, perceived effectiveness, ethicality, intervention coherence, opportunity cost, and self-efficacy. A theoretically informed generic questionnaire was proposed to assess these constructs across healthcare settings [9]. It comprises seven five-level Likert item questions with each measuring one construct of the TFA.

### Sample size estimation

This study was nested in a larger study that aimed to determine the acceptability of HIV self-testing services and preference of models for delivering HIVST testing services among AGYW. The parent study enrolled 377 participants between December 2024 and May 2025 [22, 25]. However, recommendations based on number of questionnaire items show that at least 5–10 participants per item for EFA and 5–20 participants per item for CFA is sufficient [26, 27]. With TFA-based questionnaire of seven items the sample size is at least 35–140 participants to cover both analyses. In this study we used all the 377 participants.

### Data collection tools and procedures

The data was collected using a pre-tested structured questionnaire that captured the socio-demographic and behavioral characteristics of the AGYW as well as the measurement of acceptability of HIVST using the TFA [22]. The generic questionnaire developed by Sekhon and colleagues (2022) [28], was used to develop customized questions to measure acceptability of HIVST, with a guiding statement like “*How much do you agree or disagree that…*” to help the participant in using the Likert scale. Each of the seven constructs of the TFA was assessed using one 5-level Likert item question weighted 1 to 5 where a higher score represents high acceptability (supplementary file1). However the construct of ethicality was not reverse coded and a question on overall acceptability was not considered, as recommended in the original questionnaire [28]. The questionnaire was administered by trained research assistants from 1st December 2024 to 31st May 2025, and the responses captured in the Kobo collect toolkit (https://www.kobotoolbox.org/). To assess willingness to use HIVST, participants were asked “If the HIVST services were available to you, on a scale of 0 to 10, where 0 means you can never use them and 10 means you will most definitely use them, how likely would you plan or be willing to use HIVST services?”. To assess the perceived HIV risk, participants were asked “On a scale of 0 to 10, where 0 means “no risk of getting HIV” and 10 means “a very high risk of getting HIV”, how would you grade your risk of getting HIV in the past 6 months?”.

### Data analysis

The data analysis was conducted in STATA version 17.0 (Texas, USA). The descriptive statistics were used to summarize the data. Continuous variables were summarized using the median and interquartile range (IQR), while the categorical variables were summarized using frequencies and percentages. The validation of acceptability scores measured using the TFA was assessed using three approaches, that is, exploratory factor analysis (EFA), confirmatory factor analysis (CFA), convergent validity and divergent validity. To conduct the exploratory factor analysis, the data was first assessed for suitability of factor analysis using the correlation matrix, the Kaiser-Meyer-Olkin (KMO) measure of sampling adequacy and the Bartlett’s test of Sphericity. For the correlation matrix, the threshold was to observe correlation coefficients ≥ 0.3 in the matrix. For the KMO, the cutoff was set at 0.6 and the significance of the Bartlett’s test was set at P value < 0.05. All the TFA-based questionnaire items for measuring acceptability of HIVST were in the same direction and there was no missing data. The number of factors to extract was guided by the scree plot – a plot of Eigen values, where the point of inflection indicates the number of factors to extract. Principal factor extraction was used and the pattern matrix was rotated using the Oblimin orthogonal method, and the rotated pattern matrix was reported. Following EFA, CFA was performed within a structural equation modelling (SEM) framework to test the hypothesized factor structure (generated by EFA) using the maximum likelihood estimator (MLE). A unidimensional model was specified in which seven items were modeled as indicators of a single latent acceptability construct. Standardized factor loadings and their 95% confidence intervals (CIs) were examined to assess the strength of the relationship between each item and the latent construct. Model fit was evaluated using multiple indices, including the root mean square error of approximation (RMSEA), comparative fit index (CFI), Tucker-Lewis index (TLI), and standardized root mean square residual (SRMR). Given that the indicator items were measured using ordinal Likert scale responses, additional analyses were conducted using generalized SEM (GSEM) to account for the ordinal nature of the data. GSEM models were estimated using both the probit and ordinal logit link functions, with the observed indicator items specified as ordinal outcomes of the latent acceptability construct. Model comparison using Akaike Information Criterion (AIC) and Bayesian Information Criterion (BIC) was used to identify the best fitting specification. The ordinal logit model was retained for final interpretation based on improved model fit. In building the GSEM ordinal logit model, the loading of “affective attitude” (anchor indicator) was fixed to “1” to scale the latent construct, and the log-odds coefficients and their 95% CIs were presented. For convergent validity, the participants were asked how willing they were to accept HIV self-testing services if offered on a scale of 0–10, where a higher score represented more willingness. This score was compared with the acceptability scores arising from the sum of weights of the seven questions used to assess the constructs of the TFA using the Pearson correlation. The assumption was that the two scores should be highly correlated with Pearson correlation coefficient value of ≥ 0.7. For divergent validity, the participants were asked to rate their perceived risk of acquiring HIV on a scale of 0–10, where a higher score represented a high HIV risk. This score was compared with the acceptability scores arising from the sum of weights of the seven questions used to assess the constructs of the TFA using the Pearson correlation. The assumption was that perceived HIV risk is a totally different construct from the acceptability of HIV self-testing, and therefore they shouldn’t be correlated. A Pearson correlation coefficient of < 0.3 demonstrated divergent validity. The reliability of the TFA questionnaire was assessed using the reliability coefficient (Cronbach’s alpha) to evaluate the extent to which the seven items consistently measured acceptability of HIVST. An alpha coefficient of ≥ 0.7 was considered acceptable. The item-total correlations and alpha values if an item was deleted were examined to assess the contribution of individual items.

## Results

### Socio-demographic characteristics of study participants

The median age of the participants was 20 years (IQR; 18, 22), and about a third were still in school (34.5%). Majority of the AGYW (70.8%) were Christians, and about a quarter lived with their primary partners (27.3%). Close to half of AGYW (47.8%) were in sexual relationship with a partner older than them by ten or more years, 41.6% were employed and the median monthly income was US dollars $42.9 (IQR: 28.6–57.4) (Table 1).

### Behavioral characteristics

Among the 377 participants, 82.8% had ever tested for HIV, 73.7% had tested within the past 12 months, and 43.2% had ever been pregnant. In the six months preceding the study, 13.3% had consistently used condoms, 29.2% reported to have multiple sexual partners and more than half (55.2%) reported to have had a sexually transmitted infection (STI). In the six months preceding the study, 11.7% of participants reported anal sex, 4.5% reported sharing injections, and 30.0% reported engaging in sexual intercourse in exchange for money or gifts. Additionally, 15.4% reported using illicit drugs before sexual intercourse. Use of HIV prevention methods was low, with only 6.6% and 5.7% of participants reporting the use of post-exposure prophylaxis (PEP) and pre-exposure prophylaxis (PrEP), respectively (Table 2).

### Exploratory factor analysis (EFA)

**Figure 1:**
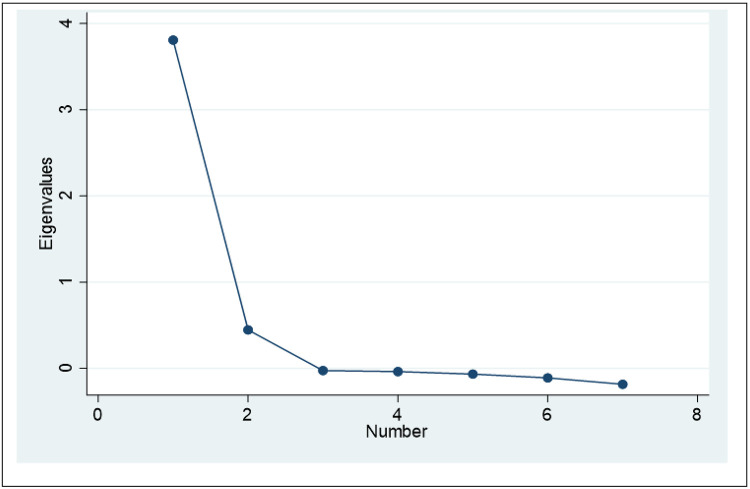
Scree plot of Eigen values

The data had a KMO of 0.88 and the Bartlett’s test of Sphericity was statistically significant (P value < 0.001). The scree plot showed three factors to be extracted (Fig. 1).

### Extraction of factors (rotated factor solution)

On rotation of the factor loadings, all the variables load significantly on one factor (factor 1) with the loadings ranging from 0.64 to 0.82 (Table 3)

### Confirmatory factor analysis (CFA)

#### Structural equation modelling (SEM)

The global model fit indices for the SEM were; RMSEA of 1.224, CFI of 0.173, TLI of −0.103, SRMR of 0.078 and the coefficient of determination (CD) was 0.917.

The standardized factor loadings ranged from 0.72 to 0.807, of which the highest were observed for perceived effectiveness (0.81), intervention coherence (0.80) and affective attitude (0.80), while ethicality showed the lowest (0.72). All the factor loadings were significant with P values < 0.001 (Table 4)

#### Generalized structural equation modelling with ordinal logit model

The GSEM yielded positive and significant log-odds coefficients for all domains. The strongest associations were observed for coherence (1.15) and perceived effectiveness (1.08), followed by opportunity cost (0.88) and ethicality (0.74). slightly lower associations were observed for burden (0.67) and self-efficacy (0.65). All estimated coefficients were significant with P values < 0.001 (Table 5)

#### Convergent and divergent validity

**Figure 2:**
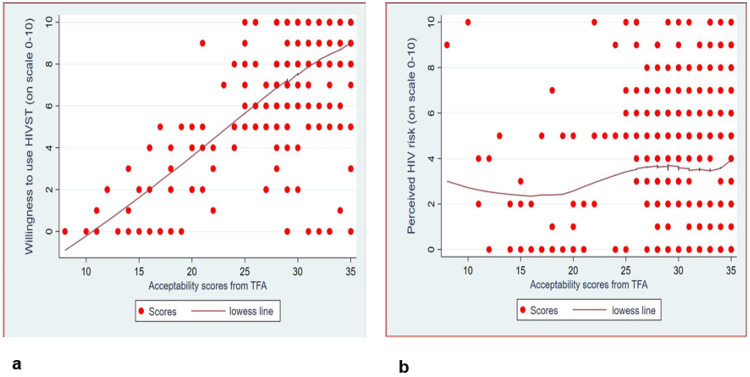
a: Scatter plot for acceptability score from TFA and willingness to use HIVST b: Scatter plot for acceptability score from TFA and perceived HIV risk

The Pearson correlation coefficient between the acceptability score from the seven constructs of TFA and the perceived willingness to accept HIV self-testing was 0.7 (Fig. 2a). The Pearson correlation coefficient between the acceptability score from the seven constructs of TFA and perceived HIV risk was 0.1 (Fig. 2b)

#### Reliability of the TFA questionnaire

The seven item TFA acceptability scale had an overall internal consistency (Cronbach’s alpha) of 0.889. The item-test and item-rest correlations were all > 0.3 (recommended threshold). The removal of any individual item did not result in a higher alpha value (Table 6).

## Discussion

This study assessed the construct validity and internal consistency reliability of the TFA-measured acceptability scores among AGYW. The findings demonstrate that the seven TFA items function as a coherent and reliable measure of acceptability in this sub-population. EFA revealed a dominant single latent factor, which supports a unidimensional TFA-theorized acceptability. Furthermore, the CFA findings provided mixed evidence on construct validity where on one had the very strong and significant factor loadings in the SEM model indicate that each construct is highly correlated with the underlying acceptability construct while on the other hand, the poor global fit indices indicate that the hypothesized one-factor model does not adequately reproduce the observed covariance structure. However, the GSEM model that accounts for the ordinal nature of the TFA domain variables showed that the latent acceptability scores were positively and significantly associated with all the seven TFA domains which indicates that higher acceptability corresponds to higher levels across each domain. The direction and magnitude of the associations were consistent with the SEM findings, thus demonstrating strong and coherent relationships between the latent acceptability construct and observed indicator items Evidence of construct validity was further observed through strong convergent validity with willingness to use HIVST and weak divergent validity with perceived HIV risk. Internal consistency reliability was excellent and all items contributed meaningfully to the scale. Collectively, these findings support the validity and reliability of the TFA-measured acceptability scores among AGYW.

### Validity of the TFA-measured acceptability scores

Although the TFA is conceptually organized into seven sub-domains, the EFA in this study identified a single dominant latent factor. This aligns with the theoretical underpinning of the TFA that all the seven constructs collectively measure one overarching domain of acceptability. Evidence from previous validation studies shows that in some settings fewer factors are extracted as compared to the theorized seven sub-domains. For instance, in the development of a digital health acceptability tool, Haydon and colleagues (2023) found that while the TFA informed the item generation, the responses primarily clustered into two broad dimensions (attitude and perceived capacity dimensions) rather than the seven sub-domains [28]. Similarly, another study on a telephone-facilitated health coaching intervention in Sweden identified fewer factors than the theoretical seven with items clustering under three main factors (affective attitude, coherence and burden) [29]. These two studies deviate from the theory behind the TFA by Sekhon and colleagues [1]. In our study, much as we report poor global fit indices of RMSEA, CFI and TLI that don’t meet the established SME fit criteria of acceptable model fit of < 0.06, ≥ 0.95 and ≥ 0.95 respectively [30], we note that the use of continuous SEM with ordinal Likert scale data may have violated distribution assumptions thus leasing to inflated misfit indices as previously reported [31]. Nonetheless, the SRMR value (0.078) falls within the acceptable limits based on recommended threshold of ≤ 0.08 [30], suggesting that absolute residual differences are relatively small. This pattern has been reported in literature particularly in large samples or when analyzing ordinal data [31]. The use of an ordinal logit link in the GSEM provide better representation of the Likert scale responses, addressing the limitations of treating ordinal variables as continuous. The GSEM findings in our study showed that all the latent acceptability scores were positively and significantly associated with all the seven TFA domains, which reinforces the construct validity of the TFA-measured acceptability scores. The consistency in direction and strength of these associations suggests that the domains operate as coherent indicators of acceptability, even when modeled using an ordinal framework. The consistency between the SEM and GSEM findings suggest that the poor global fit in the SEM may be partly attributable to modelling assumptions rather than true construct validity. Taken together, the findings from the EFA, SEM and the GSEM support the construct validity of the TFA-measured acceptability scores.

Further validity testing showed evidence of convergent validity with the acceptability score strongly correlated with the participants’ willingness to use HIVST. Methodological standards suggest that a correlation coefficient of ≥ 0.7 is considered evidence of strong convergence between constructs that should in theory be related [32, 33]. Thus, the observed correlation in our study supports the assertion that the TFA is indeed capturing the underlying construct of acceptability. In contrast divergent validity was also supported by the weak correlation between the acceptability score and perceived HIV risk. The weak correlation in this study therefore, demonstrates that the TFA measure is not merely reflecting general perceptions but specifically capturing the intervention acceptability. Findings from other TFA validation studies further support this interpretation. For example, the higher acceptability scores were significantly associated with participants’ willingness to engage in health coaching [29]. Similarly, in a study aimed at developing a digital health tool, the acceptability scores were strongly related to self-reported intension to use telehealth service, while showing weak correlation with unrelated psychological constructs [28]. This supports both convergent and divergent validity. The observed convergent and divergent validity in our study further confirm that the TFA scale is conceptually coherent and provides empirical evidence of its construct validity of acceptability scores. This strengthens confidence in the TFA’s applicability for assessing the acceptability of health interventions like HIVST among AGYW in low-resource settings.

### Internal consistency reliability of the TFA

The TFA acceptability scale demonstrated good internal consistency reliability in measuring acceptability scores with a strong Cronbach’s alpha. All items exhibited strong item-test and item-rest correlations, suggesting that each construct contributed meaningfully to the overall scale without redundancy. Importantly, removal of any individual item did not improve the Cronbach’s alpha thus supporting retention of all seven TFA sub-domains in the final TFA-based measurement of acceptability. According to psychometric standards, Cronbach’s alpha of > 0.8 reflects strong internal coherence of multi-item scales assessing latent constructs [12, 17, 34]. Similar levels of internal consistency have been reported in other studies applying TFA-informed measures of acceptability across health interventions [6, 29]. This further supports the reliability of the framework when operationalized quantitatively. Therefore, these findings provide evidence that the TFA-based measurement of acceptability is reliable for measuring health intervention acceptability among AGYW in low-resource settings.

### Limitations

This study has some limitations that should be considered when interpreting the findings. First, the data were cross sectional, which limits the ability to assess how acceptability may change over time. Secondly, all the measures relied on self-reported data which may be subject to recall and social desirability bias, particularly given the sensitivity of HIV-related behaviors, however, the period of recall was limited to six months apart from the period of past HIV testing (12 months). Thirdly, the study participants were selected using a non-probability sampling technique which may have introduced selection bias. The study focused on AGYW in urban settings, and while this is a key population for HIV prevention, the results may have limited generalizability to other age groups, gender or settings. Nonetheless, the systematic biases highlighted may have had minimal influence on the findings.

## Conclusions

This study provides empirical evidence supporting the construct validity and reliability of acceptability scores measured using the TFA among AGYW in Uganda. The seven TFA-informed items collectively formed a single latent acceptability construct and demonstrate strong convergent validity with willingness to use HIVST, weak divergent validity with perceived HIV risk, and strong internal consistency reliability. The scale may serve as a useful quantitative tool for measuring intervention acceptability among AGYW in similar low-resource settings.

## Supplementary Material

This is a list of supplementary files associated with this preprint. Click to download.

• Supplementaryfile1.pdf

## Figures and Tables

**Table 1:** Socio-demographic characteristics of AGYW enrolled in the study

Variable	Categories	Frequency (N = 377)	Percentage (%)
Age (years)	Median (IQR)	20 (18, 22)	
Schooling status	In School	130	34.5
	Not in school	247	65.5
Religion	Christians	267	70.8
	Moslems	107	28.4
	No religion	3	0.8
Age difference with any partner	< 10 years	197	52.2
	≥ 10 years	180	47.8
Live with primary partner	Yes	103	27.3
	No	274	72.7
Employment status	Employed	157	41.6
	Not Employed	220	56.4
Monthly income (USD)*	Median (IQR)	42.9 (28.6–57.4)	

* Exchange rate at the time of writing: 1 USD ≈ 3500 Uganda Shillings (UGX).

**Table 2:** Behavioral characteristics of AGYW enrolled in the study

Variable	Category	Frequency (N = 377)	Percentage (%)
Ever tested for HIV	Yes	312	82.8
	No	65	17.2
Had HIV test in past 12 months*	Yes	230	73.7
	No	82	26.3
Ever pregnant	Yes	163	43.2
	No	214	56.8
Consistent condom use in past six months	Yes	50	13.3
	No	327	86.7
Had more than one sexual partner	Yes	110	29.2
	No	267	70.8
Had STI in past six months	Yes	208	55.2
	No	169	44.8
Shared injections in past six months	Yes	17	4.5
	No	360	95.5
Had anal sex in past six months	Yes	44	(11.7)
	No	333	(88.3)
Had sex in exchange of money/gifts in past six months	Yes	113	30.0
	No	264	70.0
Used illicit drugs before sex in past six months	Yes	58	15.4
	No	319	84.6
Used PEP in past six months	Yes	25	6.6
	No	352	93.4
Used PrEP in past six months	Yes	20	5.7
	No	357	94.7

* N = 312

**Table 3:** Rotated solution of factor loadings (pattern matrix) and unique variances

Indicator item	Factor 1	Factor 2*	Uniqueness
Affective attitude	0.75		0.43
Burden	0.64	0.49	0.35
Self-efficacy	0.64	0.48	0.35
Ethicality	0.69		0.51
Opportunity cost	0.74		0.45
Effectiveness	0.82		0.33
Coherence	0.82		0.32

* Factor loadings of < 0.3 were suppressed

**Table 4:** The standardized factor loadings of the indicator items estimated by Structural Equation Modelling model

Indicator item	Standardized factor loading (95% CI)	P value	Residual variance (95% CI)
Affective attitude	0.80 (0.76, 0.83)	< 0.001	0.37 (0.32, 0.42)
Burden	0.77 (0.73, 0.80)	< 0.001	0.41 (0.36, 0.47)
Self-efficacy	0.77 (0.73, 0.80)	< 0.001	0.41 (0.36, 0.47)
Ethicality	0.72 (0.68,0.76)	< 0.001	0.48 (0.43, 0.55)
Opportunity cost	0.79 (0.76, 0.82)	< 0.001	0.38 (0.33, 0.43)
Effectiveness	0.81 (0.78, 0.84)	< 0.001	0.35 (0.31, 0.40)
Coherence	0.80 (0.77, 0.83)	< 0.001	0.36 (0.31, 0.41)

**Table 5:** The log-odds coefficients for the indicator items in a GSEM model

Indicator item	Log-odds coefficients (95% CI)	P value
Affective attitude	1	< 0.001
Burden	0.67 (0.49, 0.84)	< 0.001
Self-efficacy	0.65 (0.48, 0.83)	< 0.001
Ethicality	0.74 (0.53, 0.96)	< 0.001
Opportunity cost	0.88 (0.65, 1.12)	< 0.001
Effectiveness	1.08 (0.76, 1.39)	< 0.001
Coherence	1.15 (0.82, 1.48)	< 0.001

**Table 6:** Internal consistency reliability of the TFA acceptability scale

Item	Item-test correlation	Item-rest correlation	Average inter-item covariance	Alpha
**Affective attitude**	0.795	0.706	0.522	0.870
**Burden**	0.768	0.657	0.518	0.877
**Ethicality**	0.720	0.627	0.519	0.880
**Intervention coherence**	0.803	0.729	0.539	0.868
**Opportunity cost**	0.787	0.702	0.533	0.870
**Perceived effectiveness**	0.807	0.734	0.537	0.867
**Self-efficacy**	0.768	0.659	0.519	0.877
**Overall scale**			0.534	0.889
